# Impact of Market Authorization Holder (MAH) system on pharmaceutical innovation in China

**DOI:** 10.1080/20523211.2025.2551794

**Published:** 2025-09-04

**Authors:** Shengyan Zhai, Quan Wang, Zheng Zhu, Runzhi Han, Yumeng Lv, Chengxi Zhu, Ruining Zhang, Lei Zhang, Li Yang

**Affiliations:** a School of Public Health, Peking University, Beijing, People’s Republic of China; b Saw Swee Hock School of Public Health, National University of Singapore, Singapore; c School of Nursing, Peking Union Medical College, Beijing, People’s Republic of China; d Engineering Research Center of Modern Preparation Technology of TCM of Ministry of Education, Shanghai University of Traditional Chinese Medicine, Shanghai, People’s Republic of China; e Bloomberg School of Public Health, Johns Hopkins University, Baltimore, MD, USA

**Keywords:** Market Authorization Holder, pharmaceutical industry, difference in difference, quasi-experimental study, innovation

## Abstract

**Background:**

The Market Authorization Holder (MAH) system in China, which separates marketing authorisation from production authorisation, was piloted in 2016 across 10 provinces and cities and was fully implemented at the end of 2019 with the new revision of the ‘Drug Administration Law of the People's Republic of China’. This study examines the impact of the MAH system on innovation in China’s pharmaceutical industry.

**Methods:**

Using data from A-share pharmaceutical enterprises between 2009 and 2023, this study employs a Propensity Score Matching Difference-in-Difference (PSM-DID) design. The sample includes 134 Chinese listed enterprises, with 73 in the treatment group and 61 in the control group.

**Results:**

The PSM-DID model, based on 1,310 observations, yields a significant DID coefficient of 0.5394. Enterprise size negatively correlates with R&D investment (−0.640), while Tobin’s Q (0.153) and Top10 (0.0104) positively correlate with R&D. The MAH policy significantly reduces financing constraints (−3.556), and management incentives positively moderate the impact of the MAH system (0.0121).

**Conclusion:**

The findings suggest that the MAH system significantly enhances pharmaceutical innovation. Management incentives strengthen this effect, while financing constraints serve as an intermediary. The MAH system effectively stimulates innovation in the pharmaceutical industry, with management incentives playing a critical moderating role.

## 1. Background

The Marketing Authorization Holder (MAH) system is a pivotal reform in the pharmaceutical industry, designed to separate marketing authorisation from production authorisation (C. Wang, 2016). While established for decades in the EU, US, and Japan, China adopted this system more recently to align with international standards. Core similarities include holding the MAH (not the manufacturer) liable for drug safety, efficacy, and quality, and enabling non-manufacturers (e.g. research institutions) to hold authorisations (Yongfa Chen et al., 2015). Key differences emerge in implementation: China initially restricted MAH eligibility to manufacturers, only expanding to Research & Development institutions via pilot programmes (2015–2019) before full legal enactment, whereas Western systems historically granted broader eligibility. China’s reforms explicitly emphasise boosting domestic R&D innovation – a focus less pronounced in mature systems. By allowing entities to market drugs without manufacturing facilities, China MAH reduces capital barriers, stimulates R&D investment, and encourages specialisation across the pharmaceutical value chain (e.g. via Contract Manufacturing Organizations) (DiMasi et al., 2003).

In China, the MAH system was introduced as part of broader national drug reforms. The State Council launched a pilot programme in 2015, allowing drug research and development institutions to assume the role of the MAH. This pilot was extended in 2018, and in 2019, the MAH system was officially incorporated into the Drug Administration Law of the People's Republic of China. By 2019, 3,239 drug products had been approved under the system, marking its success in encouraging innovation (National Medical Product Administration, 2019).

In recent years, researchers have analyzed the MAH system in China from different angles. Pre-pilot research established its necessity and feasibility (Shao et al., 2014), while post-implementation studies highlight its impact. R&D institutions and non-manufacturers (especially in oncology) drive new drug development, freeing firms to invest in innovation rather than production (Han & Zhang, 2019; Ren et al., 2016; L. Wang, 2016). Pilots in Shanghai and Jiangsu improved industrial concentration, management efficiency, and CMO partnerships (Bai et al., 2019). The system streamlines approvals and influences technology transfer dynamics (Dong, 2019; Yifei Chen & Jin, 2020). Over 1,100 MAH applications by 2018 included significant participation from R&D institutions (Han & Zhang, 2019).

Collectively, these studies confirm the MAH system unlocks R&D potential by clarifying stakeholder responsibilities, reducing redundant infrastructure, lowering entry barriers, and mitigating financial constraints – particularly critical in pharma given high R&D costs and uncertainty.

Prior evidence indicates that external financing critically influences enterprises’ ability to pursue R&D and product innovation (Hall & Lerner, 2010). Pharmaceutical firms face heightened challenges due to high initial costs and innovation-related uncertainties, rendering them susceptible to financial constraints (DiMasi et al., 2003). The MAH system addresses this by separating product commercialisation rights from production obligations. This reform allows research-oriented entities to prioritise innovation investments while mitigating manufacturing-related capital burdens, thereby attracting financiers who value innovation-centric models.

Pharmaceutical innovation is inherently resource-dependent, requiring sustained investments in financial, technological, and human capital across extended R&D timelines (12–14 years) characterised by high costs and uncertainties (Hu & Yuan, 2022). When coupled with performance-linked rewards (e.g. innovation profit-sharing, equity-based compensation), MAH system facilitates managerial engagement in high-risk R&D activities. This synergistic effect improves resource allocation efficiency, ultimately strengthening drug development pipelines through sustained R&D investments.

Building on these insights, we propose the following hypotheses:

H1: The MAH pilot has a positive effect on the innovation of pharmaceutical enterprises.

H2: The MAH pilot enhances the innovation performance of pharmaceutical enterprises by alleviating their financing constraints.

H3: Management incentives positively moderate the impact of the MAH system on innovation.

This study aims to fill this gap by quantitatively evaluating the innovation outcomes driven by the MAH system in China. Through this analysis, we seek to provide a deeper understanding of the system’s role in stimulating pharmaceutical innovation and shaping the industry’s future.

## 2. Methods

### 2.1. Data

This study uses unbalanced panel data from listed pharmaceutical enterprises in China from 2009 to 2023. Data sources include financial reports, the CSMAR database, and the Wind database. The following criteria were applied to ensure the rationality and validity of the sample.

1. Exclusion of Special Treatment (ST) enterprises: ST enterprises – designated by the China Securities Regulatory Commission (CSRC) under the Shanghai and Shenzhen Stock Exchange Listing Rules (Stock Listing Rules of Shenzhen Stock Exchange [Revised in 2022] [2022]) – are companies experiencing consecutive annual losses or exhibiting abnormal financial conditions (e.g. negative net assets, audit disclaimers). These firms face heightened delisting risks and operational instability (Exchange, 2022). Their exclusion mitigates distortion in innovation metrics, as financial distress may force R&D budget cuts or strategic pivots unrelated to MAH policy effects.
2. Exclusion of non-R&D firms: enterprises without R&D activities (e.g. pure distributors, marketing-focused firms) were excluded. This aligns with the MAH system’s core objective to stimulate R&D-driven innovation by entities like research institutions. Non-R&D firms inherently lack the capacity to respond to MAH incentives, making their inclusion irrelevant to this study’s focus.
3. Exclusion of samples with missing key variables to maintain data continuity and reliability.
4. Exclusion of extreme outliers to mitigate undue influence on results.

All continuous variables were winsorized at the 1st and 99th percentiles to reduce outlier impacts.

### 2.2. Study design

The Difference-in-Differences (DID) model is widely used in policy evaluation to assess the causal impact of interventions by comparing changes in outcomes over time between treatment and control groups (Plantinga, 2021). However, the traditional DID model may suffer from sample selection bias and heterogeneity. To address these limitations, Propensity Score Matching (PSM) (Rosenbaum & Rubin, 1983) is often integrated with DID. PSM constructs a control group with characteristics similar to the treatment group, balancing pre-treatment covariates and reducing selection bias (Wang et al., 2019). The combined PSM-DID approach enhances the robustness of causal inference and has become increasingly prevalent in policy evaluation studies (Xu et al., 2024).

This study focuses on the ten provinces and cities that implemented the MAH system pilot in 2016, using the Propensity Score Matching-Difference in Differences (PSM-DID) method to analyze data from Beijing, Shanghai, Tianjin, Hebei, Jiangsu, Zhejiang, Fujian, Shandong, Sichuan, and Guangdong. The basic regression model is a multidimensional fixed-effect model, formulated as follows (model 1):

$$\mathrm{RN}D_{\mathrm{it}}={\text{β}}_{0}+{\text{β}}_{1}\mathrm{Trea}t_{i}+{\text{β}}_{2}\mathrm{Pos}t_{i}+{\text{β}}_{3}\mathrm{Trea}t_{i\;}\times \mathrm{Pos}t_{i}+\mathrm{rContro}l_{\mathrm{it}}+\tau_{i}+\mu_{t}+\epsilon_{\mathrm{it}}$$

In this model, irepresents the enterprise; t stands for year; $\mathrm{RN}D_{\mathrm{it}}\;$measures the innovation degree of the enterprise, equals to the ratio of R&D investment to total assets (RND), while the main explanatory variable is the interaction term DID($\mathrm{Trea}t_{i\;}\times \mathrm{Pos}t_{i}$), which captures the influence of the MAH system. The pilot implementation is in 2016, hence the period is divided into pre-pilot (before 2016) and post-pilot (after 2016), with the variable $\mathrm{Pos}t_{i}$ taking the value 1 for the post-pilot period and 0 for the pre-pilot period. The $\mathrm{Trea}t_{i\;}$indicates whether an enterprise is located in one of the pilot cities, with a value of 1 for pilot cities and 0 for non-pilot cities. The $\mathrm{Contro}l_{\mathrm{it}}$ are the control variables; $\tau_{i}\;$and $\mu_{t}$ are fixed effects; $\epsilon_{\mathrm{it}}$ is random error.

The Organization for Economic Cooperation and Development (OECD) formulated evaluation indicators for enterprise innovation, covering six aspects including innovation input, innovation sources, enterprise planning, innovation output, and technology transfer (K. Liu & Yang, 2015). The pharmaceutical industry is characterised by substantial R&D investment and prolonged development cycles. Critically, R&D outcomes often require multiple years to materialise. Given these inherent industry dynamics and consistent with established methodologies in pharmaceutical innovation research (Y. Liu & Gao, 2024), we choose innovation input to reflect the innovation of pharmaceutical industry in this paper, specifically the ratio of R&D investment to total assets (RND). Additionally, we have selected RID, the ratio of R&D investment to enterprise operating income in our robustness test.

Based on previous studies (Long et al., 2023; Wan et al., 2024), control variables in the model include enterprise financial indicators, such as leverage ratio (Lev), total asset turnover (ATO), and Tobin’s Q (TobinQ), as well as enterprise characteristics like size. Corporate governance factors, such as the shareholding ratio of the top 10 shareholders (Top10), board size (Board), equity restraint degree (Balance3), proportion of independent directors (Ind), and major shareholder capital occupation (Occupy), are also included. The specific definitions of each variable are shown in Table 1.

**Table 1.** Variable definitions.

Variable classification	Variables	Definition
Dependent variable	RND	the ratio of R&D investment to total assets
	RID	the ratio of R&D investment to enterprise operating income
Company scale	Size	Company size, the natural logarithm of total assets
	Board	Board size, the natural logarithm of the total number of board members
Financial status	Lev	The leverage ratio, the ratio of total liabilities to total assets, can reflect the health of an enterprise's capital structure
	ATO	Total asset turnover ratio, the ratio of net sales to average total assets, measures the efficiency of an enterprise in generating revenue from its assets
	TobinQ	Tobin Q, the ratio of a company's market value to its total assets
Company Governance	Indep	The proportion of independent directors, the ratio of the number of independent directors to the total number of members of the enterprise's board of directors
	Top10	The shareholding ratio of the top ten shareholders, the proportion of the total number of shares held by the top ten shareholders to the total share capital of the enterprise
	Balance3	Equity restraint degree, the ratio of the shares of the second to tenth largest shareholders to those of the largest shareholder
	Occupy	The occupation of funds by major shareholders and the proportion of capital held by major shareholders to total assets
Financing constraint	SA	SA index
Management Incentives	MShare	level of managerial share ownership

To check the robustness of the study, the dependent variable is changed to RID, the ratio of R&D investment to enterprise operating income, and the model is formulated as follows (model 2):

$$\mathrm{RI}D_{\mathrm{it}}={\text{β}}_{0}+{\text{β}}_{1}\mathrm{Trea}t_{i}+{\text{β}}_{2}\mathrm{Pos}t_{i}+{\text{β}}_{3}\mathrm{Trea}t_{i\;}\times \mathrm{Pos}t_{i}+\mathrm{rContro}l_{\mathrm{it}}+\tau_{i}+\mu_{t}+\epsilon_{\mathrm{it}}$$

For further analysis, financing constraint (Sa) may affect the incentive effect of MAH system on innovation. In this study, the mediating effect – financing constraint (Sa), was introduced into the original baseline regression model for further analysis, and the following mediating effect model was established (model 3):

$$\mathrm{RN}D_{\mathrm{it}}=\alpha_{0}+\alpha_{1}\mathrm{DI}D_{\mathrm{it}}+\alpha_{3}Sa_{\mathrm{it}}+\sum \alpha_{c}\mathrm{Contro}l_{s}+\mu i+\delta t+\epsilon_{\mathrm{it}}$$

As the above analysis, management incentives may have a positive moderating effect on the innovation incentive effect of the MAH system. The Management incentives (Mshare) was introduced into the original baseline regression model for further analysis, and the following moderating effect model was established (model 4):

$$\mathrm{RN}D_{\mathrm{it}}=\alpha_{0}+\alpha_{1}\mathrm{DI}D_{\mathrm{it}}+\alpha_{2}\mathrm{DI}D_{\mathrm{it}\;}\times \mathrm{Mshar}e_{\mathrm{it}}+\alpha_{3}\mathrm{Mshar}e_{\mathrm{it}}+\sum \alpha_{c}\mathrm{Contro}l_{s}+\mu i+\delta t+\epsilon_{\mathrm{it}}$$

## 3. Results

### 3.1. Descriptive statistics

1. Table 2 presents a preliminary comparison of key variables between the pilot group (*n* = 983) and the non-pilot group (*n* = 1029). The pilot group reported higher mean (2.809 vs. 2.205, *p* < 0.001) and median (2.284 vs. 1.713) RND values compared to the non-pilot group, with greater variability in the pilot group (Std. Dev.: 2.393 vs. 2.200). Pilot company sizes were larger on average (mean: 22.239 vs. 21.863, *p* < 0.001; median: 22.253 vs. 21.783) and exhibited slightly more dispersion in size (Std. Dev.: 1.096 vs. 0.908), and demonstrated significantly higher asset turnover (ATO: 0.583 vs. 0.529, *p* < 0.001). Significant governance differences included lower leverage (Lev: 0.315 vs. 0.332, *p* = 0.033), reduced board independence (Indep: 0.366 vs. 0.378, *p* < 0.001), larger board size (Board: 2.263 vs. 2.231, *p* < 0.001), and fewer related-party transactions (Occupy: 0.013 vs. 0.018, *p* < 0.001). No statistically significant differences existed in ownership concentration (Top10, *p* = 0.136), board power balance (Balance3, *p* = 0.319), or market valuation (TobinQ, *p* = 0.265).

**Table 2.** Preliminary comparison analysis of the variables.

	Pilot (*n* = 983)			Non-pilot (*n* = 1029)			
Variables	Mean	Median	Std. Dev.	Mean	Median	Std. Dev.	*p*-value
RND	2.809	2.284	2.393	2.205	1.713	2.200	0.000***
Size	22.239	22.253	1.096	21.863	21.783	0.908	0.000***
Lev	0.315	0.284	0.180	0.332	0.308	0.186	0.033
ATO	0.583	0.559	0.250	0.529	0.491	0.260	0.000***
Indep	0.366	0.333	0.048	0.378	0.333	0.052	0.000***
Board	2.263	2.303	0.149	2.231	2.303	0.165	0.000***
Top10	56.207	57.284	13.158	55.243	54.229	15.717	0.136
Balance3	0.900	0.745	0.664	0.931	0.703	0.735	0.319
TobinQ	2.671	2.035	1.905	2.584	2.066	1.598	0.265
Occupy	0.013	0.006	0.023	0.018	0.008	0.027	0.000***

### 3.2. Propensity score matching

To control for differences in enterprise characteristics, we apply Propensity Score Matching (PSM) using all control variables from the model 1 as covariates. A Logit model is used to estimate the propensity score, incorporating variables such as Size, Lev, ATO, Indep, Board, Top10, Balance3, TobinQ, and Occupy.

We employ nearest-neighbour matching with one neighbour and a calliper width of 0.05 to ensure similarity between matched enterprises. As a result, 134 pharmaceutical enterprises were selected, comprising 73 in the treatment group and 61 in the control group.

**Figure 1.**
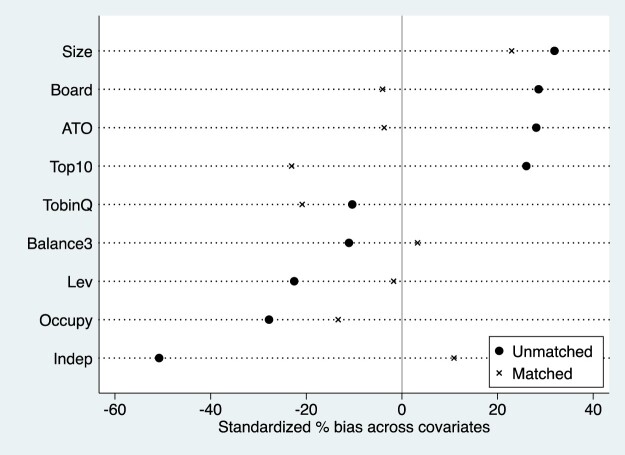
PSM balance check results.

A balance check is conducted to assess the effectiveness of matching in reducing covariate differences between groups. The results, presented in Figure 1, indicate that matching significantly reduces the mean differences and biases of most control variables, particularly for key covariates such as Board, ATO, and Lev. Through PSM, we can effectively reduce the systematic differences in key variables between the treatment group and the control group, thereby reducing the potential impact of confounding variables on the estimation of causal effects. No significant bias remains under the t-test, and reductions in pseudo R², LR chi², and bias metrics confirm that matching has successfully improved comparability between the treatment and control groups.

### 3.3. PSM-DID regression

The matched samples were reintroduced into model 1 for regression, incorporating 107 enterprise-level fixed effects (stkcd) and 14 year-level fixed effects (year) to control for unobserved enterprise-specific and time-specific factors. This isolates the true effect of the MAH policy and covariates. The PSM-DID regression results are presented in Table 3, with 1,310 observations. The F-statistics exceed 7.0, indicating statistical significance, and the adjusted R-squared value suggests the model explains over 60% of the variation in R&D investment, confirming the model's robustness. The coefficient for the MAH policy (DID) is 0.524 (*p* < 0.01) without controls and increases to 0.539 (*p* < 0.01) with controls, strongly supporting H1 that the MAH system incentivizes R&D investment. Notably, major shareholder capital occupation – operationalised as controlling shareholders diverting company funds through interest-free loans, asset transfers, or receivable manipulations – exerts a severe negative influence on R&D intensity (Occupy: −4.991, *p* < 0.01). This governance failure directly constrains innovation financing by siphoning critical capital from research pipelines. Concurrently, larger firm size associates with reduced R&D investment (Size: –0.640, *p* < 0.01), suggesting scale economies may inadvertently dilute innovation focus.

**Table 3.** Effects of the pilot MAH on the pharmaceutical innovation.

Variable	Research investment ratio (1)	Research investment ratio (2)
DID	0.524***	0.539***
	(4.18)ß	(4.35)
Enterprise size (Size)		−0.640***
		(−3.14)
Leverage ratio (Lev)		0.234
		(0.80)
Total asset turnover (ATO)		−0.158
		(−0.43)
Proportion of independent directors (Indep)		1.984
		(1.51)
Board size (Board)		0.396
		(1.16)
Shareholding ratio of the top 10 shareholders (Top10)		0.010**
		(2.08)
Equity restraint degree (Balance3)		−0.185**
		(−2.12)
Tobin's Q (TobinQ)		0.153***
		(4.11)
Major shareholder capital occupation (Occupy)		−4.991***
		(−3.52)
_cons	2.354***	14.22***
	(38.28)	(3.02)
Enterprise	Yes	Yes
Year	Yes	Yes
N	1310	1310
F	17.508***	7.062***
R^2^	0.662	0.679

Note: *p < 0.1, **p < 0.05, ***p < 0.01.

A key prerequisite for using the DID model in this study is that the pilot and non-pilot groups must meet the ‘parallel trends’ assumption. This assumption posits that both groups should exhibit similar trends in the outcome variable before the implementation of the MAH policy. To test this, an event study method is employed, using 2015 as the base period, the year immediately preceding the policy's implementation. The study examines whether there were significant differences in innovation levels between enterprises in pilot and non-pilot provinces prior to the MAH system intervention.

**Figure 2.**
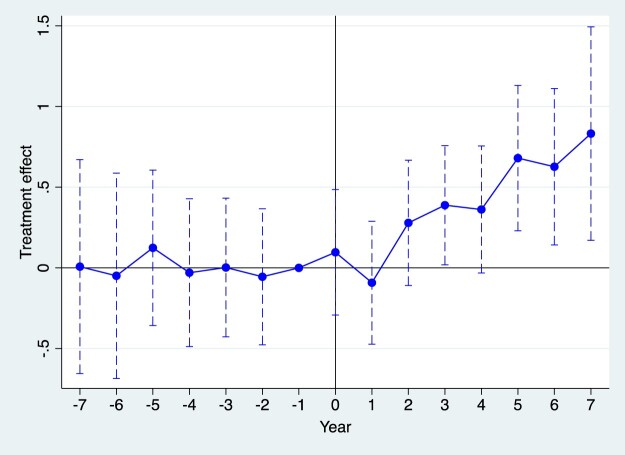
Parallel trend test result.

The results of the parallel trend test are presented in Figure 2. The coefficients for the pre-treatment periods (−7 to –1, representing 2009–2016) are not statistically significant (*p*-values > 0.1 for all periods), indicating no significant differences in pre-treatment trends between the treatment and control groups. This supports the parallel trends assumption and bolsters the credibility of the difference-in-differences (DID) estimates.

Following the implementation of the MAH pilot policy in 2016 (year 0), the point estimates turn positive as early as the first post-treatment year (year 1, 2017). However, statistically significant positive marginal effects emerge later, becoming evident in years 5–7 (2021–2023). This temporal pattern indicates that the policy did not exert an immediate significant effect but manifested a discernible positive influence on R&D investment after an approximate 5-year lag. The upward trajectory in the magnitude and significance of the policy's marginal effect over successive post-treatment years demonstrates that the MAH system has progressively stimulated pharmaceutical enterprises to increase R&D investment, fostering an environment conducive to innovation. These findings align with theoretical expectations of a time lag between complex policy interventions and observable outcomes.

By implementing model 2, the mediating effect – financing constraint (Sa), was introduced into the original baseline regression model for further analysis. As shown in Table 4, the coefficient for DID is –0.022(*p* < 0.05), indicating that after the implementation of the MAH pilot reform, financing constraints for enterprises in pilot regions decreased by 2.2%. Additionally, the coefficient for financing constraints (Sa) is −3.556, also significant at the 1% level. These results suggest that the policy intervention effectively alleviated financial constraints for firms in the pilot areas. Specifically, higher values of Sa are associated with lower R&D investment. This supports the validity of Hypothesis H2, demonstrating that the MAH system promotes innovation by easing financing constraints, thus encouraging increased R&D input.

**Table 4.** Effect of financing constraint on MAH pilot.

Variable	Financing constraint	Research investment ratio
Financing constraint (Sa)		−3.556***
		(−4.14)
DID	−0.022***	0.463***
	(−4.25)	(3.72)
Enterprise size (Size)	0.024***	−0.556***
	(3.66)	(−2.77)
Leverage ratio (Lev)	−0.028*	0.134
	(−1.91)	(0.46)
Total asset turnover (ATO)	0.0130	−0.112
	(1.11)	(−0.31)
Proportion of independent directors (Indep)	−0.0243	1.898
	(−0.45)	(1.43)
Board size (Board)	−0.055***	0.200
	(−2.76)	(0.58)
Shareholding ratio of the top 10 shareholders (Top10)	−0.001***	0.006
	(−4.81)	(1.19)
Equity restraint degree (Balance3)	0.004	−0.173*
	(0.79)	(−1.95)
Tobin's Q (TobinQ)	−0.003*	0.142***
	(−1.90)	(3.90)
Major shareholder capital occupation (Occupy)	−0.222**	−5.782***
	(−2.26)	(−3.96)
_cons	3.589***	26.98***
	(24.88)	(4.43)
Enterprise	Yes	Yes
Year	Yes	Yes
N	1310	1310
F	8.143***	8.312***
R^2^	0.972	0.683

Note: * *p* < 0.1, ** *p* < 0.05, *** *p* < 0.01.

As the above analysis, management incentives may have a positive moderating effect on the innovation incentive effect of the MAH system. By implementing model 3, the moderating effect result is presented as Table 5. The coefficient of the interaction term DID_Mshare is 0.012(*p* < 0.05). This indicates that the treatment effect of the MAH policy on firms’ R&D investment intensity increases by 0.012 percentage points for every 1-percentage-point increase in managerial shareholding (Mshare). In practical terms, the moderating variable amplifies the treatment effect, suggesting that enterprises with higher values of management incentives benefit more from the intervention in terms of innovation investment, which proves the previous hypothesis H3.

**Table 5.** Effect of management incentives on MAH pilot.

Variable	Research investment ratio
DID	0.451***
	(3.32)
DID*Mshare	0.012**
	(2.45)
Management incentives (Mshare)	0.005
	(0.90)
Enterprise size (Size)	−0.661***
	(−3.26)
Leverage ratio (Lev)	0.222
	(0.76)
Total asset turnover (ATO)	−0.179
	(−0.49)
Proportion of independent directors (Indep)	2.055
	(1.59)
Board size (Board)	0.356
	(1.02)
Shareholding ratio of the top 10 shareholders (Top10)	0.010*
	(1.67)
Equity restraint degree (Balance3)	−0.183**
	(−2.07)
Tobin's Q (TobinQ)	0.154***
	(4.13)
Major shareholder capital occupation (Occupy)	−4.871***
	(−3.48)
_cons	14.72***
	(3.09)
Enterprise	Yes
Year	Yes
N	1310
F	7.449***
R^2^	0.681

Note: * *p* < 0.1, ** *p* < 0.05, *** *p* < 0.01.

### 3.4. Robustness check

To further strengthen the robustness of this study's findings, we reanalyse the benchmark model by replacing the dependent variable, RND, with RID, the ratio of R&D investment to enterprise operating income. The PSM-DID results, presented in Table 6, show that the coefficient (0.018) for the interaction term DID variable remains positive and significant. The adjusted R² (0.537) indicates strong model fit, consistent with our primary specification.

**Table 6.** Robustness test result.

Variables	Ratio of R&D investment to enterprise operating income (1)	Ratio of R&D investment to enterprise operating income (2)
DID	0.018***	0.018***
	(4.26)	(4.15)
Enterprise size (Size)		−0.021**
		(−2.52)
Leverage ratio (Lev)		0.032**
		(2.43)
Total asset turnover (ATO)		−0.103***
		(−6.85)
Proportion of independent directors (Indep)		0.060
		(1.21)
Board size (Board)		0.008
		(0.71)
Shareholding ratio of the top 10 shareholders (Top10)		−0.000
		(−1.28)
Equity restraint degree (Balance3)		−0.004
		(−1.38)
Tobin's Q (TobinQ)		0.003**
		(2.11)
Major shareholder capital occupation (Occupy)		−0.149***
		(−2.91)
_cons	0.045***	0.530***
	(23.22)	(2.84)
Firm	Yes	Yes
Year	Yes	Yes
N	1310	1310
F	18.124***	7.178***
R^2^	0.493	0.537

*p < 0.1, **p < 0.05, ***p < 0.01.

This validates the benchmark regression results and confirms that the MAH policy robustly maintains a positive impact on pharmaceutical innovation.

### 3.5. Placebo test

**Figure 3.**
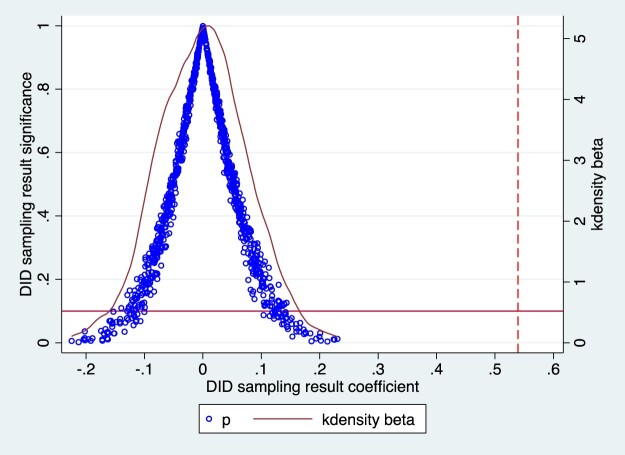
Placebo test result.

To ensure the robustness of the MAH policy’s effects, a placebo test was conducted. By randomly assigning treatment to periods or enterprises where no actual intervention occurred, we assessed whether the observed effects on RND were due to the policy or random noise. The experiment was repeated 1,000 times with random data generation to minimise interference from rare events. The scatter plot in Figure 3 shows that the regression results follow a normal distribution, and the randomly selected samples did not reflect the test area’s effects. This confirms that the findings successfully passed the placebo test.

## 4. Discussion

This investigation provides robust empirical evidence that China’s Marketing Authorization Holder (MAH) system pilot policy has significantly stimulated innovation capacity within domestic pharmaceutical enterprises. Comparative analysis between pilot and non-pilot regions revealed elevated mean and median ratios of R&D expenditures to total assets in MAH-adopting jurisdictions. These disparities may reflect the concentration of pilot zones in economically advanced regions, where institutional ecosystems and resource availability inherently foster innovation-driven growth.

Employing a quasi-experimental propensity score matching difference-in-differences (PSM-DID) framework across 134 listed pharmaceutical firms, our analysis demonstrated a statistically significant positive innovation effect attributable to the MAH policy (DID coefficient = 0.5394). Notably, firm size exhibited an inverse relationship with R&D efficiency (β = −0.640), aligning with organisational theory positing that bureaucratic inertia and complex regulatory compliance in larger entities constrain agile innovation (Hodgson, 1989). Conversely, market valuation metrics (Tobin’s Q: 0.153) and concentrated equity ownership among top shareholders (0.0104) emerged as positive determinants of R&D intensity. Elevated Tobin’s Q ratios signal market confidence in firms’ growth trajectories, incentivizing strategic reallocation of capital toward innovation to sustain competitive advantages. The shareholder concentration effect corroborates the theory (Shleifer & Vishny, 1986) that major stakeholders enhance resource coordination and risk mitigation for long-cycle R&D initiatives. In contrast, excessive ownership concentration (Balance3, –0.185) and capital misappropriation by controlling shareholders (Occupy, –4.991) significantly suppressed innovation, highlighting governance-related barriers under the MAH framework.

A critical mechanistic finding is that the MAH policy significantly reduces financing constraints, with a coefficient of –3.556, indicating that the policy's impact on innovation is partially mediated by reduced financing barriers. By expanding drug registration eligibility to research institutions and independent developers, the MAH system diversifies funding channels and catalyzes collaborative innovation networks. This structural shift has concurrently stimulated growth in ancillary sectors such as contract research organizations (CROs) and contract development/manufacturing organizations (CDMOs), thereby strengthening the pharmaceutical value chain’s resilience (Long et al., 2023). Such systemic enhancements underscore the MAH system’s role in mobilising latent financial and institutional resources toward innovation.

Furthermore, management incentives were found to positively moderate the relationship between the MAH system and innovation (coefficient = 0.012). Empirical evidence suggests that equity-based compensation and performance-linked rewards mitigate principal-agent conflicts by aligning executive priorities with long-term innovation goals (Li & Zhang, 2014). This finding counters the ‘sales-biased neglect of R&D’ phenomenon observed in firms with misaligned incentive regimes (Lv et al., 2011) emphasising the need for governance reforms to optimise innovation-oriented decision-making.

The MAH policy, first implemented in 2016 across 10 provinces and cities, has since been extended nationwide following the updated Drug Administration Law in 2019. This nationwide rollout is crucial in addressing the increasing healthcare demands in China. By stimulating pharmaceutical innovation, optimising resource allocation, and improving drug supply quality, the MAH system plays a key role in enhancing public health. However, it is important to recognise that the MAH system also imposes more stringent quality management and risk control requirements, presenting a significant challenge for small R&D companies.

### 4.1. Policy implications

Since its 2016 pilot inception and subsequent national codification under the 2019 Drug Administration Law, the MAH system has emerged as a pivotal driver of China’s pharmaceutical innovation ecosystem. To maximise its public health impact, policymakers should consider:

1. Targeted Financial Interventions: establishing state-backed financing guarantees and subsidised credit mechanisms to mitigate capital access disparities, particularly for small and medium-sized enterprises (SMEs) facing elevated funding barriers.
2. Governance Optimization: mandating diversified executive incentive schemes (e.g. equity participation, milestone-linked bonuses) to reinforce R&D prioritisation across organisational hierarchies.
3. Enhanced Regulatory Oversight for R&D-Intensive MAHs: the National Medical Products Administration (NMPA) should operationalise MAH supervision through risk-based regulatory compliance inspections to ensure the enhanced drug quality and safety.

### 4.2. Limitations and future directions

While this study advances understanding of MAH-related innovation dynamics, two limitations warrant acknowledgment. First, its reliance on R&D expenditure proxies rather than direct innovation outputs (e.g. new molecular entity approvals, patents) constrains causal inference given outcome metrics face reporting delays (Deng et al., 1999) and may not reflect R&D efficiency or strategic focus. Second, the restriction of our sample to publicly listed A-share firms inherently excludes privately held enterprises and SMEs, introducing potential selection bias. This limits the generalizability of findings to entities facing distinct capital constraints and regulatory exposure – precisely those likely to experience disproportionate MAH impacts.

Subsequent research should expand sample scope by integrating non-listed pharmaceutical firms (e.g. via NMPA licensing databases) to assess MAH’s heterogeneous effects across ownership structures and incorporate direct innovation metrics to assess the policy’s translational impact on further innovation and therapeutic advancement.

## 5. Conclusion

This study utilised PSM-DID analysis, based on open data from China A-share pharmaceutical enterprises, to assess the impact of the MAH system on pharmaceutical innovation. The results show that the MAH pilot system significantly enhanced innovation in Chinese pharmaceutical enterprises, with the impact being moderated by management incentives and mediated by financing constraints.

These findings highlight the MAH system’s key role in unlocking the R&D potential of pharmaceutical enterprises, stimulating innovation, and driving the sector's growth. The policy has encouraged increased participation from R&D institutions, diversifying the entities involved in drug development and marketing, which in turn fosters a more dynamic and innovative pharmaceutical industry.

## Authors’ contributions

SZ: Conceptualisation, Methodology, Validation, Formal Analysis, Investigation, Data Curation, Writing – Original Draft Preparation, Writing – Review & Editing; QW: Writing – Original Draft Preparation, Writing – Review & Editing; ZZ, RH, YL, CZ, RZ, LZ: Writing – Review & Editing; LY: Conceptualisation, Methodology, Resources, Supervision, Project Administration. All authors have read and agreed to the published version of the manuscript. LY, as the corresponding author, is responsible for this study.

## Consent for publication

Not applicable.

## Data Availability

The datasets used and/or analyzed during the current study are available from the corresponding author on reasonable request.
